# Phylogeny of *Calvittacus* Revealing a New Species from China (Acari: Eriophyidae)

**DOI:** 10.3390/insects13050431

**Published:** 2022-05-05

**Authors:** Yue Yin, Yi-Wen Lu, Xin-Yu Liu, Xiao-Feng Xue

**Affiliations:** Department of Entomology, Nanjing Agricultural University, Nanjing 210095, China; 2018202048@njau.edu.cn (Y.Y.); 2020102100@stu.njau.edu.cn (Y.-W.L.); 2020102101@stu.njau.edu.cn (X.-Y.L.)

**Keywords:** *Calvittacus*, Eriophyoidea, taxonomy

## Abstract

**Simple Summary:**

Eriophyoid mites (Eriophyoidea) are strictly phytophagous, consisting of over 5000 extant species, in which many species are pests. The genus *Calvittacus* includes only four species and is endemic in the Oriental Region. In this study, we combined morphological characters and molecular approaches to delimitate the *Calvittacus* species and recovered one new species, *Calvittacus spectabilus* sp. nov. The new species is vagrant on lower leaf surface, causing no apparent symptom to the host plant.

**Abstract:**

Eriophyoid mites (Eriophyoidea) are distributed worldwide and are the largest superfamily in the Acari. After over one and a half centuries of field surveys, regional fauna of eriophyoid mites remains unclear. The genus *Calvittacus* Xue, Song & Hong 2006 is endemic in the Oriental Region, including four species—*C. chenius* Xue, Wang, Song & Hong, 2009; *C. mollissimus* Han, Xue & Hong, 2017; *C. regiae* Xue, Song & Hong 2006; and *C. swidanus* Song, Xue & Hong, 2009. In this study, we describe one new species, *Calvittacus spectabilus* sp. nov., collected on *Bougainvillea spectabilis* (Nyctaginaceae) from China (the Oriental Region). Phylogenetic analysis based on mitochondrial *COI* barcode sequences confirmed the *C. spectabilus* sp. nov., coinciding with the morphological delimitation. We further discussed the potential distribution of the *Calvittacus* species and underlined the integrative approaches in eriophyoid mite delimitation.

## 1. Introduction

Eriophyoid mites (Eriophyoidea) comprise over 5000 described species [1], leading to the largest superfamily in the Acari. They are strictly phytophagous and can cause massive economic losses, e.g., *Aceria tosichella* Keifer and *A. guerreronis* Keifer [2]. Although eriophyoid mites have a worldwide distribution [3], they are supposed to be distributed mainly in temperate regions [4]. After over one and a half centuries of field surveys of eriophyoid mites, new species continue to be described in recent years. For instance, given more than 1200 eriophyoid mites described in China, an average of 12 new species per year were added in the last three years [5]. Furthermore, the Chinese fauna of eriophyoid mites was suggested, including over 2300 species [6]. It is likely that 1000 species could be recovered in China in future studies.

The genus *Calvittacus* was established by Xue et al. [7], based on the type species *C. regiae* Xue, Song & Hong 2006. It is characterized by broad dorsal annuli, forming thickened bands and a furrow; scapular setae ahead of the rear shield margin, in a centrad direction; all coxal setae present; and legs and opisthosoma with the usual setae [7]. To date, only four species have been reported in this genus; all of them infest angiosperms from China.

To understand the diversity of eriophyoid mites in China, especially the *Calvittacus* species, Xiao-Feng Xue and colleagues conducted a long-term field survey since 2002. In this study, we describe and illustrate *Calvittacus spectabilus* sp. nov. on *Bougainvillea spectabilis* (Nyctaginaceae), which was collected from south China. We further discuss the potential distribution of the *Calvittacus* species and underline the integrative approaches in describing new eriophyoid mite species.

## 2. Materials and Methods

### 2.1. Taxa Sampling and Morphological Identification

**Figure 1:**
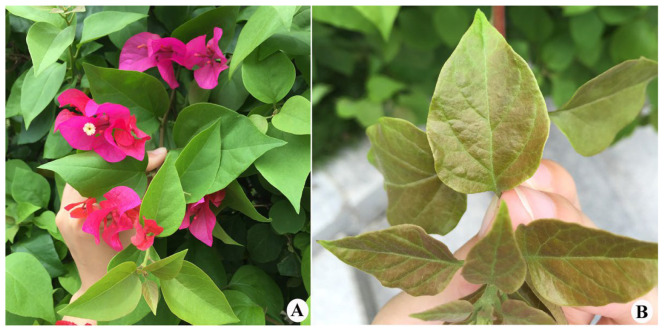
Host plant and damage symptom. (**A**) host plant (*Bougainvillea spectabilis*); (**B**) damage symptom.

Samples were collected from *Bougainvillea spectabilis* (Nyctaginaceae) in the field (Figure 1), using a hand lens (30×), in China. Mite samples were stored in 95–96% ethanol at −20 °C prior to DNA extraction. Mite specimens were also slide-mounted using Keifer’s Booster and a modified Berlese medium [8], but without adding additional fibers as was suggested by de Lillo et al. [9]. The morphological terminology used herein follows Lindquist [10] and Amrine et al. [11], internal female genitalia nomenclature follows Chetverikov [12], and the generic classification is made according to Amrine et al. [11], in combination with descriptions of all the published genera after 2003. Specimens were measured following de Lillo et al. [9]. They were examined with the aid of a Zeiss A2 (Germany) research microscope with phase contrast, and semi-schematic drawings were made. Microphotographs were taken with a Zeiss A2 (microphoto camera AxioCam MRc) research microscope with phase contrast or differential interference, using 10× eyepieces at 100× oil magnification, connected to a computer using Axiovision image analysis software. For each species, the holotype female measurements precede the corresponding range for paratypes (given in parentheses). For males, only ranges are given. If no variation was observed among measurements, it will be indicated with an “*”. All measurements are in micrometres (μm) and represent lengths, when not otherwise specified. The holotype and seven paratypes are deposited in the Arthropod/Mite Collection of the Department of Entomology, Nanjing Agricultural University (NJAU), Jiangsu Province, China [13].

### 2.2. DNA Extraction and Sequencing

In total, 14 individuals collected from three locations (Baxianshan, Fujian; Wanlu, Hainan; Haikou, Hainan) were selected for molecular analysis. Genomic DNA was extracted using a DNeasy Blood and Tissue Kit (Qiagen) following a modified protocol [14]. PCR was used to amplify the 658 bp barcode region of the mitochondrial *COI* gene using the primer pairs bcdF01/bcdR04 [14]. PCR reaction, purification, and sequencing followed Yin et al. [6].

### 2.3. Species Delimitation

Fourteen *COI* sequences were aligned and trimmed in Geneious 8.1.9 [15]. All sequences were blasted in GenBank and checked for possible contaminants. All the sequences were deposited in GenBank under accession numbers: MZ482549-MZ482557, OM892490-OM892494. In addition, collection data, taxonomic information, sequences, and trace files were submitted to BOLD [16] under number: AEC8688. We constructed a Neighbor-Joining tree that employed the K2P distance metric and used the Taxon ID tree tool on BOLD. Genetic distances were calculated with MEGA 6.0 [17] employing the Kimura 2-parameter (K2P) distance parameter [18].

### 2.4. Phylogenetic Analysis

The Eriophyoidea includes three families, i.e., Phytoptidae, Eriophyidae, and Diptilomiopidae [11]. To test the phylogenetic position of *Calvittacus spectabilus* sp. nov. within Eriophyoidea, we constructed an additional data matrix including five species of Phytoptidae (i.e., *Trisetacus ehmanni*, *Trisetacus thujivagrans*, *Trisetacus juniperinus*, *Boczekella fabris*, *Setoptus koraiensis*), nine species of Eriophyidae (i.e., *Aculus ichnocarpi*, *Phyllocoptes taishanensis*, *Surapoda tianlinensis*, *Aculus populi*, *Leipothrix sabinae*, *Tetra zhouzhis*, *Tegolophus ulmi*, *Abacarus floridulus*, *Epitrimerus sabinae*), and seven species of Diptilomiopidae (i.e., *Apodiptacus rubi*, *Diptilomiopus nobilus*, *D. bischofiae*, *Quadracus cudraniae*, *Rhyncaphytoptus mori*, *R. celtis*, *Trimeroptes luanchuanensis*). *Osperalycus tenerphagus* and *Gordialycus* sp. from the family Nematalycidae were used to root the tree because they were suggested to be the sister group of Eriophyoidea [19,20]. All *COI* sequences were aligned using MAFFT v7.2 [21]. The best-fit DNA sequence evolution model for our data was selected using the Bayesian information criterion (BIC) in jModelTest ver. 2.1.1 [22] with the GTR+G+I model selected for *COI*. Phylogenetic analyses were conducted using maximum likelihood (ML) and Bayesian inference (BI). ML analyses were performed using nucleotide sequences in the RAxML-HPC2 on XSEDE (3.2.3) [23] implemented in CIPRES Science Gateway V. 3.3 [24], using nonparametric bootstrap with 1000 replicates for node support. BI analyses were computed with MrBayes 3.2.6 [25], using two separate data partitions for codons (1st+2nd, 3rd). Two parallel runs of four independent chains were conducted for 10 × 10^7^ generations with samples every 1000 generations. The first 25% of the samples were discarded as burn-in.

## 3. Results

### 3.1. Taxonomy

Family Eriophyidae Nalepa
Subfamily Phyllocoptinae Nalepa
Tribe Phyllocoptini Nalepa
Genus *Calvittacus* Xue, Song & Hong

**Figure 2:**
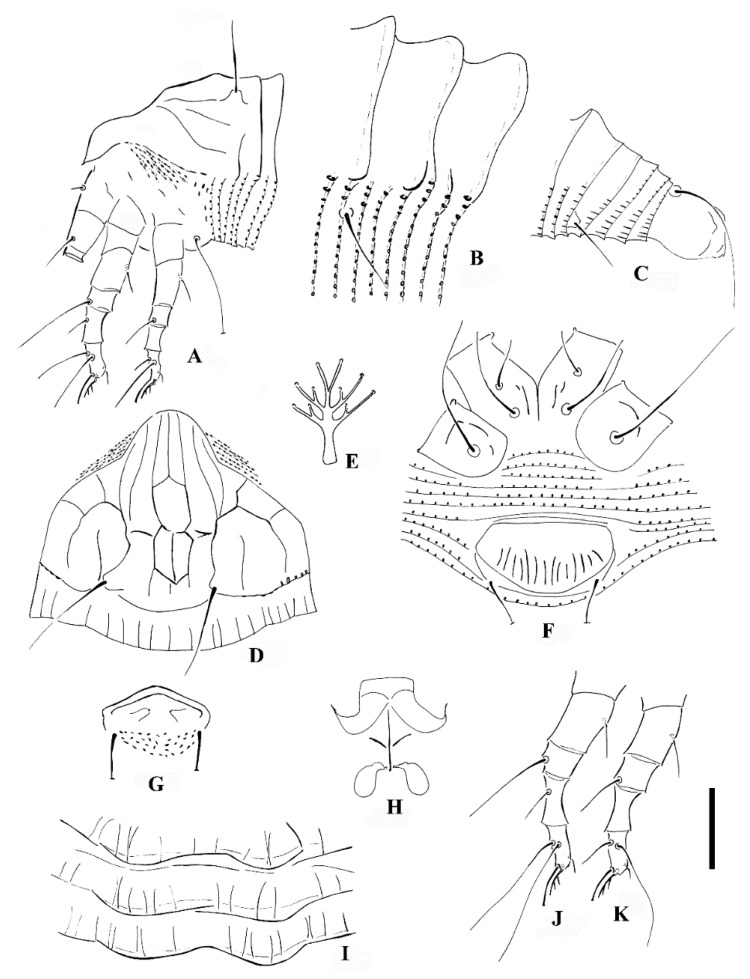
*Calvittacus spectabilus* sp. nov. (**A**) lateral view of anterior part of body; (**B**) lateral microtubercles; (**C**) lateral view of telosoma; (**D**) prodorsal shield; (**E**) empodium; (**F**) female coxigenital area; (**G**) male external genitalia; (**H**) female internal genitalia; (**I**) dorsal annuli; (**J**) leg I; (**K**) leg II. Scale bar: 18 μm for (**A**,**C**); 15 μm for (**D**,**F**–**H**,**J**,**K**); 4 μm for (**B**,**E**,**I**).

**Figure 3:**
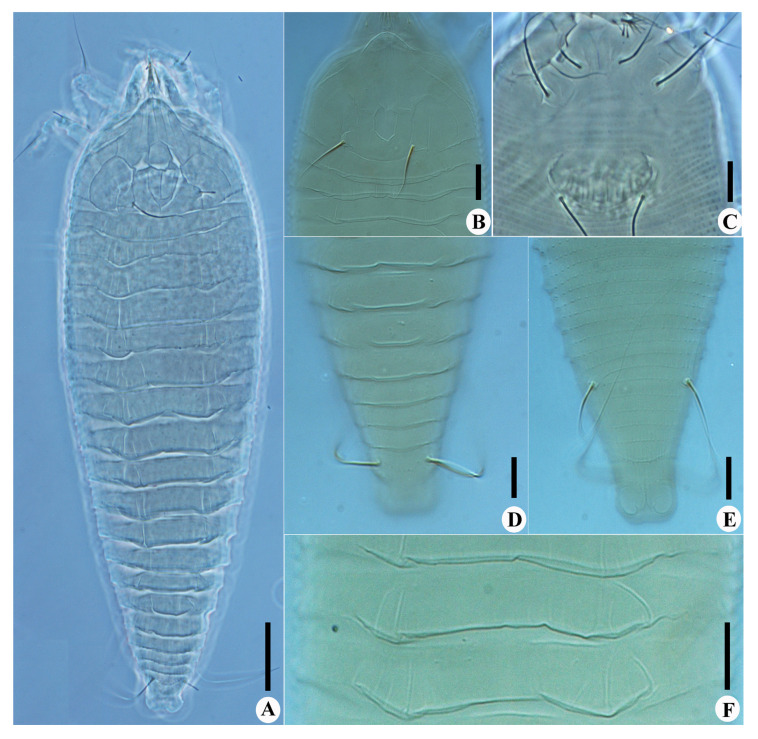
*Calvittacus spectabilus* sp. nov. (**A**) dorsal view; (**B**) prodorsal shield; (**C**) female coxigenital area; (**D**) dorsal view of telosoma; (**E**) ventral view of telosoma; (**F**) dorsal annuli. Scale bar: 20 μm for A; 10 μm for (**B**–**F**).

**Figure 4:**
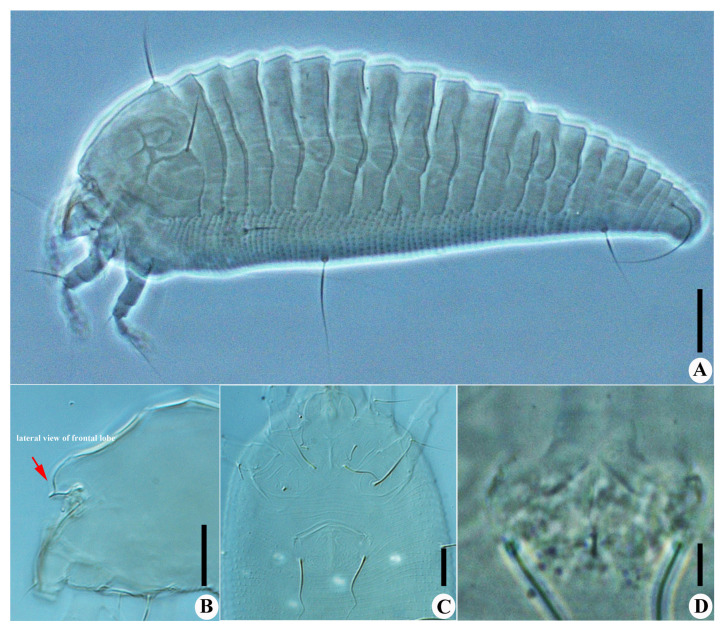
*Calvittacus spectabilus* sp. nov. (**A**) lateral view; (**B**) lateral view of gnathosoma; (**C**) male coxigenital area; (**D**) female internal genitalia. Scale bar: 20 μm for A; 10 μm for (**B**,**C**); 5 μm for (**D**).

*Calvittacus spectabilus* sp. nov. (Figure 2, Figure 3 and Figure 4).

Description. Female (n = 29): Body fusiform, 191 (182–205), 64 (58–65) wide, 65 (63–67) thick; light yellow in colour. Gnathosoma 16 (16–17), projecting downwards, cheliceral stylets 14 (14–15), pedipalp coxal seta (*ep*) 3 (2–3), dorsal pedipalp genual setae (*d*) 8 (7–8), palp tarsal ventral setae (*v*) absent. Prodorsal shield 41 (37–45), including the frontal lobe, 56 (52–56) wide, frontal lobe broad (Figure 2D and Figure 3B), with a spine in lateral view (Figure 4B); median line discontinuous, present at anterior 1/3 and posterior 1/3, admedian lines connected with median line by short transverse lines, forming a large cell at center and two small cells at posterior, submedian lines complete. Scapular tubercles ahead of rear shield margin, setae *sc* 22 (21–22), 21 (20–21) apart, projecting centered. Coxigenital region with 6* semiannuli between coxae and genitalia, smooth; coxal plates with granules, anterolateral setae on coxisternum I (*1b*) 7 (7–8), 13 (13–14) apart; proximal setae on coxisternum I (*1a*) 15 (15–18), 9 (8–9) apart; proximal setae on coxisternum II (*2a*) 32 (32–38), 24 (22–24) apart. Prosternal apodeme present, 9*. Leg I 28 (28–30), femur 10 (9–10), basiventral femoral setae (*bv*) 10 (10–11); genu 4 (4–5), antaxial genual setae (*l*″) 21 (20–21); tibia 6*, paraxial tibial setae (*l*′) 5 (5–6), located at basal 1/3; tarsus 6*, paraxial fastigial tarsal setae *ft*′ 15 (15–17), antaxial fastigial tarsal setae *ft*″ 18 (18–19), setae *u*′ 4 (4–5); tarsal empodium (*em*) 6 (6–7), simple, 3-rayed, tarsal solenidion (*ω*) 7*, rod-like. Leg II 25 (25–27), femur 8 (8–10), basiventral femoral setae (*bv*) 10 (10–12); genu 4 (4–5), antaxial genual setae (*l*″) 8 (6–8); tibia 6*; tarsus 5*, paraxial fastigial tarsal setae *ft*′9 (8–9), antaxial fastigial tarsal setae *ft*″ 21 (19–21), setae *u*′ 4*; tarsal empodium (*em*) 6*, simple, 3-rayed, tarsal solenidion (*ω*) 7*, rod-like. Opisthosoma dorsally with 18 (18–19) semiannuli, with elliptical granules on ridges, with three ridges, middorsal ridge ended in a broad furrow; ventrally with 71 (69–71) semiannuli, with elliptical to linear microtubercles. Setae *c2* 16 (16–17), on ventral semiannulus 14 (14–15), 61 (59–61) apart; setae *d* 31 (29–31), on ventral semiannulus 26 (26–27), 40 (40–42) apart; setae *e* 14 (14–20), on ventral semiannulus 43 (41–43), 20 (19–20) apart; setae *f* 18 (18–24), 20 (20–21) apart, on 5th ventral semiannulus from rear; setae *h1* absent, setae *h2* 50 (45–50). Female genitalia 13 (13–14), 25 (22–25) wide, coverflap with 12 to 14 ridges, setae *3a* 40 (39–40), 18* apart. Internal genitalia: spermathecae ovoid, oriented posterolaterad; spermathecal tubes relatively short; transverse genital apodeme trapezoidal.

Male (n = 9): Body fusiform, 153–181, 56–58 wide; light yellow in colour. Gnathosoma 14–15, projecting downwards, cheliceral stylets 13*, pedipalp coxal seta (*ep*) 3*, dorsal pedipalp genual setae (*d*) 6*, palp tarsal ventral setae (*v*) absent. Prodorsal shield 42–45, including the frontal lobe, 50–52 wide. Scapular tubercles ahead of rear shield margin, setae sc 18–20, 18–20 apart, projecting centrad. Coxigenital region with 4 semiannuli between coxae and genitalia, smooth; coxal plates with short lines, anterolateral setae on coxisternum I (*1b*) 5–6, 12* apart; proximal setae on coxisternum I (*1a*) 11*, 8–9 apart; proximal setae on coxisternum II (*2a*) 22–25, 21–22 apart. Prosternal apodeme present, 8*. Leg I 26–28, femur 9*, basiventral femoral setae (*bv*) 10–13; genu 4–5, antaxial genual setae (*l*″) 15–20; tibia 6*, paraxial tibial setae (*l*′) 5*, located at basal 1/3; tarsus 5*, paraxial fastigial tarsal setae *ft*′ 15*, antaxial fastigial tarsal setae *ft*″ 17–19, setae *u*′ 4*; tarsal empodium (*em*) 5–6, simple, 3-rayed, tarsal solenidion (*ω*) 6–7, rod-like. Leg II 25–28, femur 8*, basiventral femoral setae (*bv*) 10–11; genu 5*, antaxial genual setae (*l*″) 6–7; tibia 5–6; tarsus 5*, paraxial fastigial tarsal setae *ft*′ 6–7, antaxial fastigial tarsal setae *ft*″ 16–19, setae *u*′ 4*; tarsal empodium (*em*) 5*, simple, 3-rayed, tarsal solenidion (*ω*) 6*, rod-like. Opisthosoma dorsally with 17–18 semiannuli, smooth, with three ridges, middorsal ridge ended in a broad furrow; ventrally with 65–70 semiannuli, with elliptical to linear microtubercles. Setae *c2* 15–17, on ventral semiannulus 14*, 52–53 apart; setae *d* 26–28, on ventral semiannulus 26*, 33* apart; setae *e* 14–15, on ventral semiannulus 41–42, 16–17 apart; setae *f* 18–20, 17* apart, on 5th ventral semiannulus from rear; setae *h1* absent, setae *h2* 38–45. Male genitalia 11*, 19* wide, setae *3a* 28–30, 15* apart.

Type material. Holotype, female (slide number NJAUFJ2.1; marked Holotype), found on *Bougainvillea spectabilis* Willd. (Nyctaginaceae), Baxianshan Park, Jinjiang, Quanzhou city, Fujian province, China, 24°30′48″ N, 118°24’37” E, elevation 251 m, 15 August 2015, coll. Yan Dong, deposited as slide-mounted specimen in the Arthropod/Mite Collection of the Department of Entomology, NJAU. Paratypes, five females on five slides and two males on two slides (slide number NJAUFJ2.2–NJAUFJ2.8; marked Paratypes), from *Bougainvillea spectabilis* Willd. (Nyctaginaceae), same details as holotype, deposited as slide-mounted specimens in the Arthropod/Mite Collection of the Department of Entomology, NJAU.

Other material. Eight females on Eight slides and three males on three slides (slide number NJAUQ314.1–NJAUQ314.11; marked Paratypes), from *Bougainvillea spectabilis* Willd. (Nyctaginaceae), Wanlu Park, Haikou city, Hainan province, China, 20°02’48” N, 110°18’47” E, elevation 10 m, 27 May 2019, coll. Yue Yin and Liang-Fei Yao, deposited as slide-mounted specimens in the Arthropod/Mite Collection of the Department of Entomology, NJAU; fifteen females on fifteen slides and four males on four slides (slide number NJAUQ330.1–NJAUQ330.19; marked Paratypes), from *Bougainvillea spectabilis* Willd. (Nyctaginaceae), Volcano Geological Park, Haikou city, Hainan province, China, 19°55’54” N, 110°13’06” E, elevation 140 m, 29 May 2019, coll. Yue Yin and Liang-Fei Yao.

Deposited as slide-mounted specimens in the Arthropod/Mite Collection of the Department of Entomology, NJAU.

GenBank accession numbers. MZ482551 (NJAUFJ2); MZ482550, MZ482553-MZ482555 (NJAUQ314); MZ482554, MZ482552, MZ482549, OM892490-OM892494 (NJAUQ330).

BOLD number. AEC8688.

Relation to the plant host. Vagrant on lower leaf surface. No apparent symptom to the host plant was observed. We suppose that the bronze in colour for the upper leaf surface of young leaves was normal for the host plant, while not induced by mites (Figure 1B).

Etymology. The specific designation *spectabilus* is derived from the species name of the host plant, *spectabilis*, changing postfix -*is* to -*us*; masculine in gender.

Differential diagnosis. The new species is morphologically similar to four *Calvittacus* species (Table 1), but can be differentiated by median, admedian and submedian lines present on the prodorsal shield (simple prodorsal shield design with few lines in the other four *Calvittacus* species), 18 dorsal annuli (11 to 13 annuli in *C. chenius*, *C. mollissimus*, and *C. regiae*; 23 annuli in *C. swidanus*), and empodium 3-rayed (empodium 5-rayed in the other four species).

Key to species of *Calvittacus*

1. Coxal plates smooth………………………………………………………………………2

-. Coxal plates with granules and short lines……………………………………………..3

2. Dorsal opisthosoma with 11 annuli, the first 7 annuli form large bounds……………

……………………………………………….. *C. chenius* Xue, Wang, Song & Hong, 2009

-. Dorsal opisthosoma with 23 annuli, the first 19 annuli form large bounds…………

………………………………………………………*C. swidanus* Song, Xue & Hong, 2009

3. Dorsal opisthosoma with 11 to 13 annuli, median or admedian lines present………4

-. Dorsal opisthosoma with 18 annuli, median, admedian and submedian lines present

……………………………………………………………………..*C. spectabilus* sp. nov.

4. Ventral annuli with round microtubercles, dorsal annuli smooth……………………

…………………………………………………… *C. mollissimus* Han, Xue & Hong, 2017

-. Ventral annuli with spiny microtubercles, dorsal annuli with filamentous micro-

tubercles………………………………………………*C. regiae* Xue, Song & Hong 2006.

### 3.2. Genetic Distance and Phylogenetic Analysis

Fourteen *COI* sequences of *C. spectabilus* sp. nov. from three populations were nearly identical in composition, with an intraspecific distance (K2P) ranging from 0.000 to 0.001. We tried to sequence the *COI* sequences of *C. chenius* and *C. swidanus*, but failed, possibly due to DNA degradation of samples, which were collected for over 10 years and were kept in 75% ethanol at room temperature. We therefore included three *Calvittacus* species in the phylogenetic analyses.

**Figure 5:**
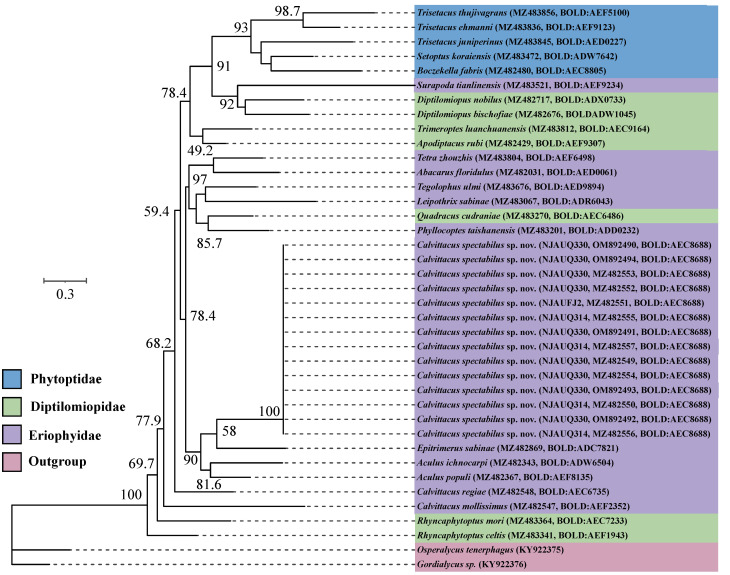
Maximum likelihood (ML) tree for *Calvittacus* species based on mitochondrial *COI* barcode nucleotide sequences.

**Figure 6:**
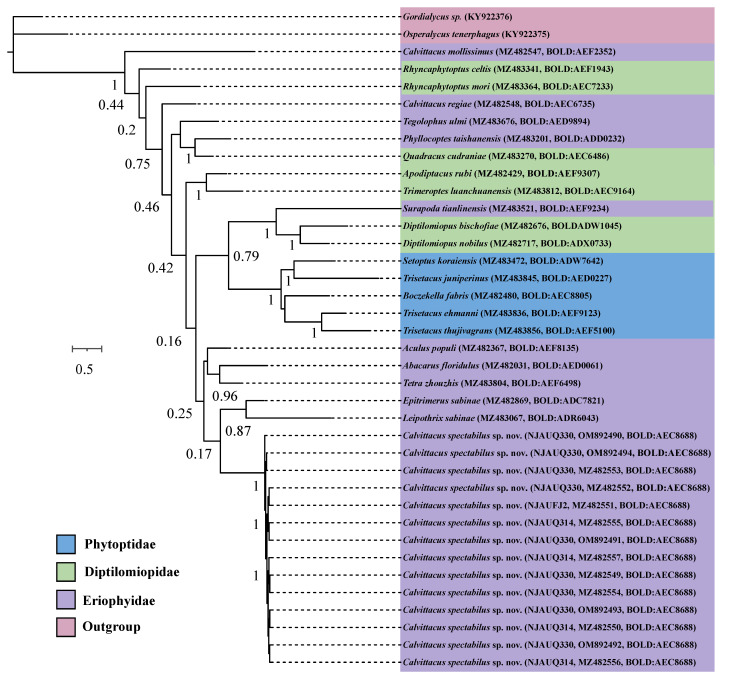
Bayesian inference (BI) tree for *Calvittacus* species based on mitochondrial *COI* barcode nucleotide sequences by codons (1st+2nd, 3rd).

The ML and BI analyses showed that *C. spectabilus* sp. nov. was nested within the Eriophyidae (Figure 5 and Figure 6), which is consistent with the morphological assignment.

## 4. Discussion

In this study, we described and illustrated one new eriophyoid mite species belonging to the genus *Calvittacus*, based on the distinct morphological characters of dorsal annuli with thickened bands, forming a furrow [7]. Eriophyoid mites are characterized by ringed opisthosoma, in which dorsal annuli vary in number (ranged from 10 to 80) and shape (forming ridges, furrows, or evenly rounded) [11]. However, dorsal annuli formed by thickened bands were occasionally found in genus *Achaetocoptes* [29], *Calvittacus* [7], *Johnella* [30], *Neovittacus* [26], and *Vittacus* [31]; all these genera hold few species (less than ca. 10). Furthermore, this morphological character has not been phylogenetically tested at the generic level to determine whether it is a synapomorphy. Although four species have been described in the *Calvittacus*, all generic assignments were based on morphological characters. Our phylogenetic results showed a non-monophyly of *Calvittacus*; however, this result was based on a fragment of mitochondrial *COI* gene sequences. Multiple genes from mitochondria and nuclear, especially genome sequences, should be determined in future analyses to account for the position of *C. spectabilus* sp. nov. and to unveil the monophyly of *Calvittacus*.

**Figure 7:**
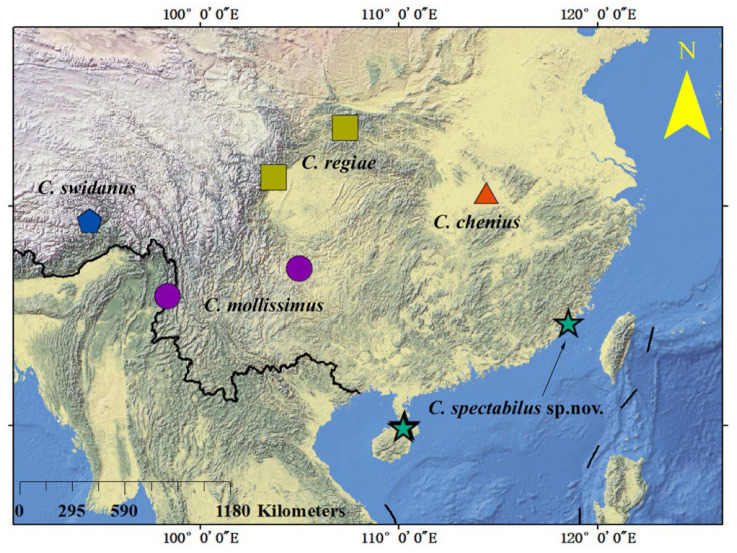
The distribution of *Calvittacus* species.

The genus *Calvittacus* was established by Xue et al. [7] based on the type species *C. regiae* Xue, Song & Hong 2006, which was collected from the Oriental Region (Figure 7). After that, three more species, collected from the Oriental Region, were assigned to this genus (Figure 7). Herein, the fifth species, *C. spectabilus* sp. nov., were also collected from the Oriental Region. Under these contexts, it is likely that the genus *Calvittacus* was endemic in the Oriental Region. However, more field surveys are warranted in future studies to understand the origin and biogeographical distribution of the *Calvittacus* species.

*COI* barcodes were recently explored in eriophyoid mite delimitation, showing a clear gap between interspecific divergence and intraspecific divergence, and thus enabling a discrimination of 99% of the eriophyoid mite species [6]. We provided 14 *COI* barcode sequences of *C. spectabilus* sp. nov., which were collected from three locations. Sequence analysis showed no intraspecific divergence. After comparing with the eriophyoid mite sequences in the Barcode of Life Data Systems database (DS-ERIYYDNA Barcodes for Eriophyoidea, available at doi: dx.doi.org/10.5883/DS-ERIYY.), all 14 sequences were assigned into one Barcode Index Number (BOLD: AEC8688), which reflects a single species; this molecular delimitation is in line with our morphological studies, confirming a new eriophyoid mite species of *C. spectabilus* sp. nov.

Simply relying upon few morphological characters to delimitate eriophyoid mites has its limitations. Inaccurate species delimitation could result from (1) species complex (e.g., *Abacarus hystrix* complex [6,32]), (2) cryptic diversity (e.g., *Diptilomiopus* species [33]), and (3) two morphological forms (protogyne and deutogyne [34]). We therefore highlight the integrative methods that combined molecular sequences (even genome sequences) and morphological characters in the description of new eriophyoid mite species.

## Figures and Tables

**Table 1:** List of *Calvittacus* species.

Species	Hosts	Distribution	Relation to Host
*C. chenius* Xue, Wang, Song & Hong, 2009 [26]	*Quercus chenii* Nakai (Fagaceae)	China	Vagrant
*C. mollissimus* Han, Xue & Hong, 2017 [27]	*Castanea mollissima* Blume (Fagaceae)	China	Vagrant
*C. regiae* Xue, Song & Hong 2006 [7]	*Juglans regia* L. (Juglandaceae)	China	Vagrant
*C. spectabilus* sp. nov.	*Bougainvillea spectabilis* Willd. (Nyctaginaceae)	China	Vagrant
*C. swidanus* Song, Xue & Hong, 2009 [28]	*Cornus macrophylla* Wall. (Cornaceae)	China	Vagrant

## Data Availability

All data is available in this paper. All sequences were deposited in the GenBank under accession numbers of MZ482549-MZ482557, OM892490-OM892494.
